# Aggression in specialized Wushu Taolu training among university students and its transfer to everyday life contexts: a qualitative study

**DOI:** 10.3389/fpsyg.2026.1919112

**Published:** 2026-08-18

**Authors:** Yunlong Pang, Mingyi Chu, Zhenbo Chen, Yuanyuan Feng, Yingshuai Qiu, Qi Dong

**Affiliations:** College of Physical Education, Qingdao University, Qingdao, China

**Keywords:** aggression, Chinese martial arts, emotion regulation, martial ethics, sport-to-life transfer

## Abstract

**Objective:**

This study aimed to explore how a contextual aggressive state is experienced, elicited, and regulated in Wushu Taolu, a routine-based discipline within Chinese martial arts (Wushu), among university students, and how participants perceived its possible transfer to everyday life. Rather than examining whether Wushu Taolu training increases or decreases aggression, the study focused on the specific form and meaning of this state within the training context and on participants’ perceptions of whether its associated effects extended into everyday life.

**Method:**

A qualitative design was adopted. Eighteen university students majoring in Wushu Taolu at a college of physical education were recruited through purposive sampling. Data were collected through three focus group interviews and three individual interviews. The interview data were transcribed verbatim and analyzed using thematic analysis with the support of NVivo 15.

**Results:**

Participants understood the phenomenon examined in Wushu Taolu training not as real hostility or violent impulse, but as a contextual aggressive state grounded in the offensive and defensive meanings of movement and embodied through gaze, force production, vocalization, rhythm, and overall bearing. This state was elicited through mental imagery and body-based cues. Participants distinguished between “aggressive experience” and “aggressive desire.” The former was regarded as a legitimate training state, whereas the latter involved an actual intention to attack or harm others and required regulation through coaches, peers, self-awareness, and martial ethics. Regarding transfer, participants generally did not perceive direct behavioral transfer into everyday life, but some reported possible associated effects such as residual arousal, faster reactions, and identity-related or stylistic influences.

**Conclusion:**

The contextual aggressive state identified in Wushu Taolu training should be understood as a temporary, instrumental, and bounded training state rather than as a stable aggressive trait. University Wushu education should guide students to understand the training meaning of this state, regulate its boundaries, and prevent it from developing into an actual desire or intention to attack or harm others or into inappropriate behavior.

## 1 Introduction

Aggression is not determined solely by stable individual traits but is activated, assigned meaning, and constrained within specific social environments. Group norms, role expectations, power relations, peer interactions, coach feedback, institutional rules, and environmental cues influence how individuals interpret external stimuli, regulate emotion and arousal, and choose between aggressive and non-aggressive responses (Allen et al., 2018). Accordingly, similar forms of heightened arousal, attack orientation, or forceful expression may carry different meanings across social contexts. Within clearly defined task goals and normative boundaries, they may serve temporary functions; once detached from the relevant context or directed toward actual others, they may develop into inappropriate aggressive intentions or behaviors. Research on aggression should therefore move beyond comparisons of higher or lower aggression levels and examine how social environments shape the generation, functions, and boundaries of aggression-related states. Sport training represents one such structured social environment, in which coaches, peers, rules, and training culture may both elicit particular states and regulate their expression and transformation (Lane et al., 2011; Jekauc et al., 2021; Robazza et al., 2023; Tamminen et al., 2024).

Wushu Taolu provides a distinctive context for examining this process. Although Taolu movements are performed in prearranged sequences, they retain offensive and defensive meanings and require practitioners to express combat intention through gaze, force production, vocalization, rhythm, and overall bearing (International Wushu Federation, 2024, 2026; Zhou, 2000). Compared with real confrontation, Taolu training retains an imagined opponent, attack direction, and combat meaning but involves neither direct physical contact nor an actual target of harm. It therefore helps distinguish combat intention and performance-related arousal from an actual intention to attack or harm others. In this study, the central phenomenon is defined as a “contextual aggressive state”: a temporary state formed around training tasks and expressed through embodied action without involving an actual intention to harm. Accordingly, aggression is used in this study as the broader psychological construct, whereas “contextual aggressive state” refers to the specific form identified within Wushu Taolu training. It differs from trait aggression, which reflects a relatively stable disposition; anger, which is an affective state; hostility, which involves antagonistic cognition; violent impulse, which entails an urge to harm; competitive intensity, which refers to the degree of effort and involvement in competition; and performance-related arousal, which may accompany but does not itself constitute this state.

Direct research on aggression or prosocial characteristics among Wushu Taolu practitioners remains scarce, and relevant evidence comes mainly from broader martial arts, combat sports, and competitive sport. A meta-analysis by Harwood et al. (2017), covering 12 studies and 507 children and adolescents, found that martial arts interventions were associated with reductions in externalizing problems. However, a systematic review of nine studies by Lafuente et al. (2021) concluded that the overall effects of martial arts and combat sports on anger and aggression remained unclear. Huang et al. (2026) further found that 84 competitive athletes reported lower total aggression and lower scores on several aggression dimensions than 106 non-athletes. These findings indicate that structured training does not necessarily increase off-field aggression, but they cannot be directly generalized to Wushu Taolu practitioners. More importantly, existing studies have mainly compared aggression levels and have rarely explained how aggression-related states are generated, function, and regulated within actual training.

Wushu Taolu cannot fully reproduce the physiological and interpersonal conditions of real physical confrontation. Taolu training lacks an unpredictable opponent, direct threat, reciprocal attack, and immediate risk of injury; therefore, the arousal and pressure experienced during practice cannot be equated with those arising in actual confrontation. Research comparing official karate competition with simulated combat has likewise identified differences in activity patterns and certain physiological responses (Chaabène et al., 2014). The value of selecting Wushu Taolu therefore lies not in treating it as a substitute for real confrontation, but in using its structured and non-contact characteristics to distinguish combat intention, performance-related arousal, embodied expression, and an actual intention to harm. Accordingly, the present study does not infer participants’ physiological or behavioral responses during real confrontation from their experiences in Taolu training.

The participants in this study were university students specializing in Wushu Taolu at a college of physical education. For these students, specialized training is embedded in university life, including classrooms, dormitories, and peer interactions. States and expressive patterns developed during training may therefore be reinterpreted and regulated in everyday interactions and may be perceived by participants as having continuing effects. Guided by instrumental emotion regulation theory (Lane et al., 2011; Jekauc et al., 2021), the distinction between surface and deep acting (Hochschild, 1983; Grandey, 2000), and sport-to-life transfer theory (Kendellen and Camiré, 2019; Tanious et al., 2023), this study addresses three questions: how university students understand and embody contextual aggressive states in Wushu Taolu training; how these states are elicited and regulated through coaches, peers, self-awareness, and martial ethics education; and how participants understand the possible transfer of these states or their associated effects into everyday life contexts. By addressing these questions, the study moves beyond comparisons of higher or lower aggression and examines the contextual generation, boundary regulation, and perceived cross-contextual influence of aggression-related training experiences.

## 2 Literature review

### 2.1 The contextual characteristics of Wushu Taolu training

Wushu Taolu is not merely a performance with no offensive or defensive implications. Instead, it is a series of movement exercises based on the principles of combat and offensive and defensive techniques. According to the International Wushu Federation, “Taolu” is a mode of practice composed of prearranged and continuously connected movements, and the choreography should reflect offensive and defensive principles while also containing hand and leg techniques, jumps, sweeps, turns, stances and footwork, balance, and qinna (International Wushu Federation, 2026). This indicates that Taolu training possesses competitive and performative features, yet its movement organization and stylistic requirements have not departed from combat, but are shaped by technical regulations, stylistic expression, and offensive and defensive meanings.

Therefore, performing the movements in Wushu Taolu training is not enough; the training must go beyond movement performance. Practitioners are also required to convey the offensive and defensive meanings embedded in the movements and the stylistic characteristics of different Wushu styles through their gaze, rhythm, force production and overall bearing (International Wushu Federation, 2024; Zhou, 2000). That is, Taolu practice pays equal attention to completing movements and to constructing and expressing an overall state. These characteristics provide an important basis for examining contextual aggressive states in Wushu Taolu training. Their roles in training and their relationships to combat meaning and stylistic form should be examined rather than reduced to simple hostility or violent tendencies.

### 2.2 The elicitation of contextual aggressive states: instrumental emotion regulation and performance mechanisms

Athletes do not necessarily aim to regulate their emotions to become calmer or experience more pleasant emotions, but actively regulate them based on performance goals, as evidenced by the research on emotion regulation in sport. From the perspective of emotion theory in competitive sport, the dynamic link between emotion and performance was emphasized by Jekauc et al. (2021). Lane et al. (2011) also found that athletes sometimes actively employed strategies that led to heightened unpleasant emotions (e.g., anger, anxiety) as they believed these emotions were valuable for athletic performance. For the current study, this line of research offers a theoretical perspective for exploring whether contextual aggressive states in Wushu Taolu training reflect actual hostility or serve performance-related functions, such as expressing the stylistic properties of specific Wushu styles and overall bearing. Performance-related arousal may accompany these states but is not treated as aggression itself.

According to Grandey (2000), surface acting refers to regulating outward expression without changing inner emotional experience, whereas deep acting involves attempts to modify inner emotional experience. Gross’s (2015) process model of emotion regulation provides a broader account of how emotional responses may be regulated at different stages. Applied to Wushu Taolu training, this means that some students may first adopt the required gaze, posture, and bearing, whereas others may imagine an opponent, strengthen combat intention, and immerse themselves in the movement. Whether such processes actually occur during training, and how students understand them, must be further explored through empirical data.

### 2.3 The boundaries and regulation of contextual aggressive states: normative mechanisms in the training context

The contextual aggressive state identified in Wushu Taolu training may serve performative and instrumental functions, but it cannot be understood as existing without limits. The review by Tamminen et al. (2024) highlighted that emotional experience, expression, and regulation in sport are not merely individual processes but are co-constructed through coach demands, peers, team norms, and relational contexts. This state is not self-generated, but is constantly affirmed, strengthened, limited, and challenged by coaches, peers, and training norms. This outlook prompts the question of which forms of combat-oriented expression and performance-related arousal are considered necessary and acceptable in Wushu Taolu training, and when they are regarded as excessive.

This boundary is reinforced by traditional Wushu ethics. Documents related to the Chinese Wushu Duanwei assessment system emphasize the simultaneous development of martial ethics and Wushu etiquette and incorporate offensive and defensive skills, Wushu styles, etiquette, and moral cultivation into assessment (Chinese Wushu Association, 2024a, 2024b; Wang, 2021). The present study therefore examines how students understand contextual aggressive states, which states are deemed reasonable, which expressions are considered excessive, and how these boundaries are regulated by coaches, peers, and martial ethics.

### 2.4 The perceived transfer of contextual aggressive states and their associated effects from training to everyday life

Beyond the training setting itself, whether contextual aggressive states or their associated effects extend into everyday life is a key question that this study must address. Kendellen and Camiré (2019) conceptualized sport-to-life transfer as a process through which what is learned in sport is transferred and then applied in domains outside sport. Tanious et al. (2023), in their review of emerging adults, also showed that abilities, experiences, and coping patterns developed in sport do not necessarily remain within the sport context, but may enter learning, family, and social interaction. For university students majoring in Wushu Taolu, this suggests the possibility that performance-related arousal, combat-oriented embodied expressions, or specific modes of self-presentation formed in training may enter dormitories, classrooms, and interpersonal interactions in the form of speaking style, conflict response, emotional activation thresholds, and interactional style.

The spillover perspective suggests that experiences and behavior patterns in one domain may spread into another (Staines, 1980), while excitation transfer theory indicates that residual arousal from a prior situation may amplify emotions and responses in a subsequent one (Zillmann, 1971, 1983). These perspectives provide theoretical resources for considering whether the effects associated with contextual aggressive states may continue outside training as short-term residual arousal or longer-term stylistic influence. Therefore, the present study treats such transfer as an empirical question, rather than assuming a direct spillover of training states into everyday life. For university students majoring in Wushu Taolu, this issue is especially important because such possible transfer does not occur in an abstract life domain, but in concrete educational settings such as classrooms, dormitories, and peer interaction.

### 2.5 Summary of the literature review

Existing studies provide insights into emotional and aggression-related issues in martial arts training, but three major gaps remain. First, findings on the effects of Wushu and combat training on anger and aggression are inconsistent, and most studies compare higher and lower levels of aggression rather than explain how contextual aggressive states are generated, used, and regulated during training. Second, Wushu Taolu has received little attention as a distinct practice with both performative and combat significance. Third, although some studies discuss whether these states or their associated effects persist beyond training, it remains unclear whether they extend into students’ everyday university life and why this process differs among students. Most importantly, these questions have rarely been explored through students’ own training experiences and interpretations of their environment.

Therefore, the current study is about the particular expression of contextual aggressive states in the training of university students majoring in Wushu Taolu, the mechanisms of their active elicitation, the boundary setting and regulation of these states, and whether and how these states or their associated effects transfer to other situations in everyday life, for instance, in classrooms, dormitories, and other interpersonal interactions. Taking this approach, the study aims to transcend the sole view of “higher” or “lower” aggression, and analyze the perceived cross-contextual transfer of these states and their associated effects with reference to the combined contexts of Wushu Taolu training and the university educational context, as well as their corresponding generative logic and regulatory mechanisms.

## 3 Participants and methods

### 3.1 Participants

The participants in this study were undergraduate students majoring in Wushu Taolu at Q University College of Physical Education. Using purposive sampling (Campbell et al., 2020; Dahal et al., 2024), a total of 18 participants were selected. The characteristics of the sample were carefully considered to capture variation and increase comparability across a range of factors: grade level, years of training, competition experience and training level (Dahal et al., 2024; Bouncken et al., 2026). The inclusion criteria were as follows: participants (1) were undergraduate students majoring in Wushu Taolu; (2) had received specialized Wushu training for more than 10 years; (3) could remember and describe their training experience and day-to-day experience clearly; and (4) were willing to participate voluntarily and provide prior informed consent. The exclusion criteria were as follows: (1) had discontinued regular Taolu training for more than one month because of injury or other reasons; and (2) refused to be audio-recorded or were unable to complete the full interview. All participants were undergraduate students in their first, second, or third academic year. Their basic characteristics are presented in Table 1.

**Table 1:** Characteristics of the participants (*N* = 18).

Characteristic	Participant information
Sex	Male, 9 (50%); Female, 9 (50%)
Year	First- to third-year undergraduate students
Major	Wushu Taolu
Specialized training experience	More than 10 years
Focus group interview participation	18
Follow-up individual interview participation	3

The university students majoring in Wushu Taolu were chosen for two reasons. Firstly, this group has experienced the movement requirements, stylistic presentation, modes of expression, and training norms of Taolu relatively systematically over a long period, so its members have extensive experience in Taolu practice. Secondly, they are in a special period of intensive professional training and, at the same time, receive education focused on personal development, emotion regulation, and social interaction (De Vries et al., 2021; Grossmann et al., 2023; Velert-Jiménez et al., 2025).

Using purposive sampling, one information-rich participant from each focus group was selected for a follow-up individual interview, resulting in three individual interviewees from the overall sample. The main criteria were as follows: they had relatively substantial, specific, or distinctive training experiences related to some of the themes discussed, such as “understanding of contextual aggressive states,” “active elicitation,” “boundary regulation,” or “perceived transfer to everyday life”; they differed in years of training, the main Wushu styles practiced, or competition experience, thus providing supplementary material for thematic comparison; some of the issues discussed were difficult to share fully in the group format and therefore required one-to-one interviews to obtain more comprehensive personal narratives; the participants had relatively strong reflective and expressive abilities, and they were able to reflect on and interpret their personal experiences in depth. This practice of further selecting individual interviewees from the overall sample is consistent with the common qualitative principle of using purposive sampling to identify information-rich cases (Palinkas et al., 2015; Hamilton and Finley, 2019; Dahal et al., 2024).

### 3.2 Interview design

This study used semi-structured interviews to collect data. Based on the research topic and prior consideration, focus group interviews were used as the primary method, supplemented by individual interviews. Focus group interviews helped participants engage in interactive discussion around shared training experiences and were better suited to revealing the implicit rules of emotional expression, stylistic requirements, and shared understandings of “legitimate aggressive experience” and what constituted “crossing the line” in Taolu training (Dedios-Sanguineti et al., 2025). For experiences that were more distinctive, more nuanced, or less easily expressed in a group setting, individual interviews were added to provide more in-depth personal material (Wakelin et al., 2024). This study was approved by the ethics committee of the authors’ institution. Before the formal interviews, the researcher conducted pilot interviews and made appropriate adjustments to the wording, order, and follow-up style of the questions based on participant feedback in order to improve clarity and contextual fit. Pilot interviews were conducted with two students, whose data were not included in the final analysis. Formal interviews were conducted in relatively quiet and familiar settings with minimal external disturbance. The researcher explained the purpose of the research, content of the interviews, recording arrangements, use of these data, and principles of anonymization before each interview, and made it clear that participants were free to decline to answer any question and that they could withdraw from the interview at any time. The formal interviews began only after informed consent had been obtained.

The focus group interviews were organized into three groups, with six participants in each group, and one participant from each group subsequently completed a follow-up individual interview. Each focus group and individual session was 40–90 min long. Audio recordings and field notes were used to capture the verbal content as fully as possible, together with important emotional expressions, pauses, and interactional information. The recordings were promptly transcribed verbatim after the interviews. The names of the participants and other identifying information were replaced with codes. All data were used solely for this study and securely stored by the researcher.

#### 3.2.1 Focus group interviews

To reduce conformity and mutual influence, the moderator emphasized that there were no correct answers and encouraged participants to express different views. Neutral follow-up questions were used, quieter participants were invited to contribute, and dominant speakers were appropriately redirected. When consensus emerged quickly, the moderator asked for different experiences or contrasting examples. Sensitive or divergent issues were further explored through individual interviews.

The following aspects were explored in the group interview guide. First, basic training background: years of training, main types of Taolu exercises practiced, training priorities (first priority, second priority, third priority); Second, participants’ understandings of contextual aggressive states and their embodied expressions through movement, gaze, facial expression, rhythm, force production, and overall bearing. Third, the elicitation of contextual aggressive states and their perceived functions, especially whether students who are not experiencing anger or hostility actively elicit such states and performance-related arousal in response to combat meaning, stylistic requirements, and training norms, and how this affects their training and competitive performance. Fourth, boundaries and norms—what is considered an acceptable or functional contextual aggressive state, which expressions are regarded as crossing the line, and the roles of coaches, peers, training order, and martial ethics in this process. Fifth, perceived transfer to everyday life, examining whether these training states or their associated effects carry over into dormitories, classrooms, friendships, and online communication. Sixth, summary and suggestions, used to collect participants’ overall understanding of contextual aggressive states in Wushu Taolu training and their views on issues requiring attention from schools and coaches.

#### 3.2.2 Individual interviews

**Figure 1:**
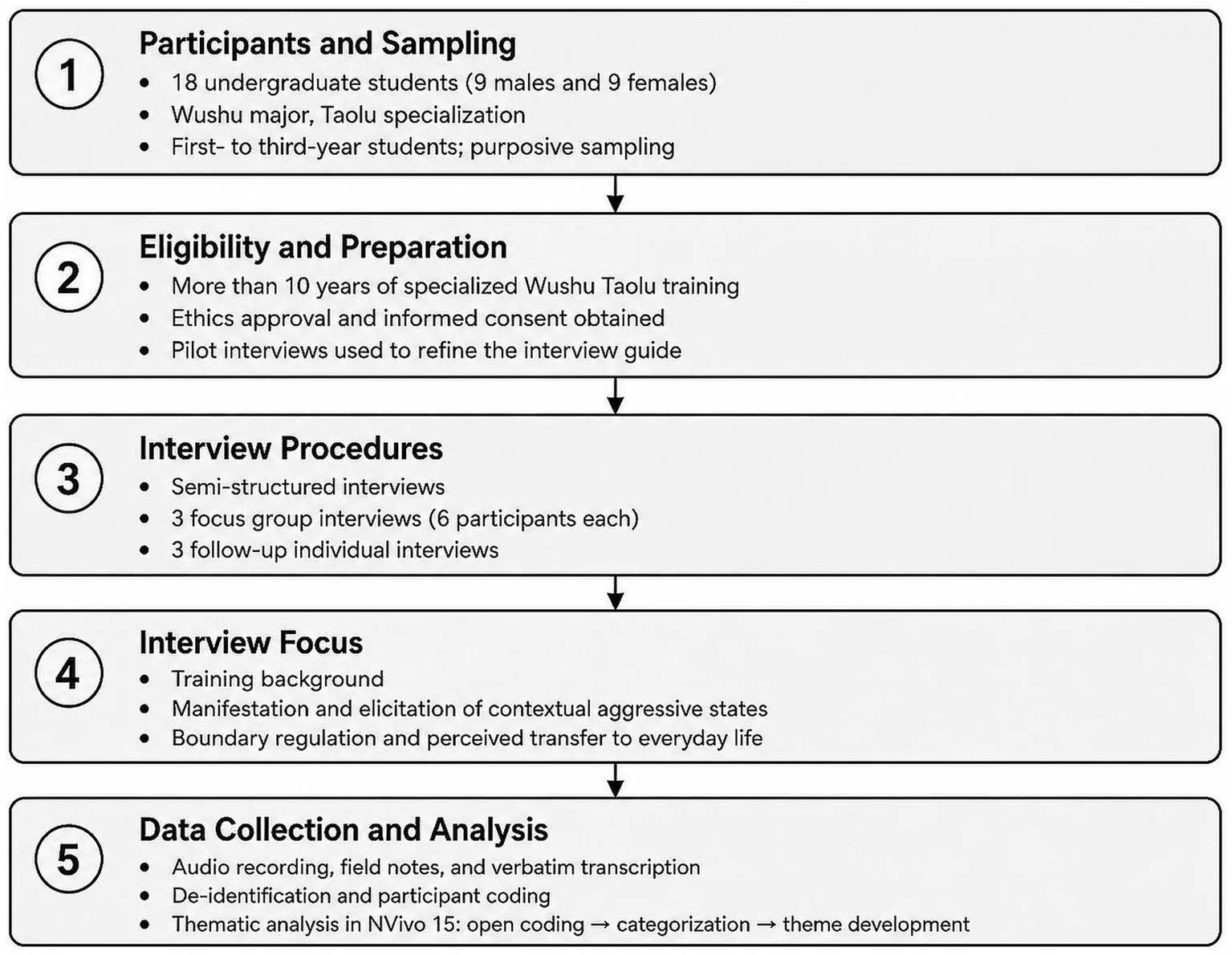
Interview design, data collection, and analysis process of the present study.

In the individual interviews, personal training experiences, personal understandings of contextual aggressive states, processes of active elicitation, functions, meanings, boundaries and regulation, perceived transfer of these states or their associated effects to everyday life, and individual differences were further explored. Specifically, the interviews focused on whether the active elicitation of contextual aggressive states (imagining an opponent, strengthening combat intention, and adjusting movements and facial expressions) further affected the participants’ speaking style, responses to conflict, emotional activation thresholds, and interpersonal interactions. In addition, individual interviews were used to explore differences in perceived transfer, regulatory capacity, training experience, and the relationships of these factors with training years, competition experience, personality traits, coaching style, and the characteristics of various Wushu styles. The overall interview design, data collection, and analysis process is summarized in Figure 1.

### 3.3 Data analysis

The interview data were subjected to thematic analysis, with NVivo 15 software used to assist with data management, coding, and theme development. The transcripts were imported into NVivo 15, where the researchers created and revised codes, organized related codes into hierarchical categories, retrieved coded excerpts, compared accounts across participants and interview types, and recorded analytic notes. NVivo was used as a data-management tool and did not automatically generate codes or themes.

Thematic analysis is ideal for exploring the relationship between individual experiences, meaning-making, and social context and enables insights to be gained into the potential generative logic, expressive boundaries, regulatory mechanisms, and other avenues through which contextual aggressive states and related emotional processes are expressed in Wushu Taolu training and are perceived to extend into everyday life contexts (Braun and Clarke, 2021; Castleberry and Nolen, 2018). The analysis was carried out as follows.

All audio recordings were transcribed verbatim, checked against the original recordings, anonymized, and imported into NVivo 15. The transcripts were subsequently read repeatedly to develop an initial understanding of the overall data.

Second, guided by the research questions, the researcher undertook open coding by assigning initial codes to meaningful words, sentences, and text segments relating to combat meaning, contextual aggressive states, performance-related arousal, emotion mobilization, training norms, boundary regulation, and perceived transfer to everyday life. The same text segment could be assigned more than one code when it contained multiple meanings.

Third, these initial codes were compared across participants and interview types. Codes with similar meanings were grouped into broader categories and organized as parent and child nodes in NVivo 15. Relationships among the categories were then examined to develop candidate themes.

Finally, the candidate themes were continually reviewed and refined against both the coded excerpts and the complete dataset. Overlapping codes were merged, unclear theme boundaries were revised, and divergent or contrasting accounts were examined rather than excluded. By combining the participants’ training and everyday life contexts, their meanings, boundaries, and interrelationships were further interpreted, ultimately leading to a systematic explanation of the research questions.

The analysis followed a primarily inductive approach, with relevant theories informing the later stages of category comparison and theme interpretation. Initial codes were generated from participants’ accounts rather than from predetermined theoretical categories. During theme development and coding, the researcher kept reflexive notes, critically explored assumptions underlying subjective interpretation, and sought to strengthen the credibility of the findings through comparisons between participants’ experiences (Byrne, 2022; Nowell et al., 2017).

## 4 From combat meaning to embodied bearing: the generation of contextual aggressive states in Wushu Taolu training

### 4.1 Contextual aggressive states grounded in the combat meaning of movement

The interview findings indicated that participants did not primarily use “aggression” in Wushu Taolu practice to refer to anger, hostility, or violent impulse. Rather, they described a contextual aggressive state embedded in movement structure, combat-related imagery, and stylistic expression.

*P1: “If I break this aggression down, I think aggression in Wushu Taolu should refer to its attack-oriented meaning, that is, what its combat meaning is. That is what aggression means to me.”*

Participants in the group interviews expressed similar views:

*If I had to summarize aggression in Wushu Taolu training in one sentence, I think it refers to the meaning of attack—that is, what the combat meaning of this movement is. This aggression is mainly reflected in one’s state during the routine, one’s gaze, and one’s control of the precision of the movements.*

*Aggression mainly lies in the fact that every movement in Wushu has its own offensive and defensive combat meaning, and that is the aggression in Wushu Taolu.*

These statements suggest that, for the participants, the contextual aggressive state is first understood through why a movement is performed in a particular way. If a punch, chopping palm, stamping step, or weapon movement is completed only in form, without an understanding of its offensive and defensive meaning, it is difficult for such a training state to emerge. P1 further pointed out that, in pure performance, “combat meaning” should even come before movement and style:

*P1: “If you are talking about pure performance, I think the three most important things should be combat meaning first, movement second, and style last. Because once you have combat meaning, your style will naturally be expressed.”*

This suggests that combat meaning is not an additional element in Taolu training but an essential component of movement quality and stylistic presentation. As demonstrated in previous studies, athletes regulate their emotions based on their performance objectives and are not solely interested in calming themselves down or inducing pleasant emotions, but rather they strategically mobilize their emotions to perform well (Lane et al., 2011; Jekauc et al., 2021; Robazza et al., 2023). In Wushu Taolu training, what participants described as “aggression” gives movement direction, purpose, and force. It is therefore better understood as an instrumental training state rather than hostility in everyday life, supporting movement execution, the communication of combat intention, and stylistic expression. Accordingly, this study uses the term “contextual aggressive state” to refer to a state grounded in combat meaning and cultivated through imagined opponents, combat intention, and embodied expression. The interview data move beyond the general statement that Taolu contains combat meaning by showing that participants recognize this state when a movement acquires an imagined target, direction, and intention.

### 4.2 Embodied expressions of contextual aggressive states

If combat meaning constitutes the internal basis of the contextual aggressive state, then gaze, force production, vocalization, rhythm, and overall bearing constitute its embodied expression. Most participants did not describe this state as a single-dimensional experience, but understood it as a comprehensive bodily state. In a group interview, one participant stated:

*I think this state is an overall temperament. It includes movement, gaze, facial expression, rhythm, and force production. These things together form aggression and overall bearing in Wushu Taolu training. None of them can be missing.*

When judging whether a person’s “state has come out,” participants particularly emphasized the importance of gaze:

*When a person’s state has come out, the first thing I look at is the eyes. I think after someone starts practicing, their eyes show their state the most. The second thing is movement and force production. It is not about how standard the movement is, but whether the movement has that feeling and is combined with force production. But whether the state is good or not can be seen from the eyes.*

Another group of participants also noted that this state was often expressed through “force production and gaze,” especially in Changquan, Nanquan, and weapon practice, where gaze direction, weapon trajectory, vocalization, and weapon sounds all strengthen this state:

*Force production and gaze are the main parts. The eyes usually need to look forward, or when using a broadsword you look at the hand and the weapon. Changquan and Nanquan movements require vocalization, and sometimes weapons also need to produce sound.*

These accounts show that the contextual aggressive state is not only internally experienced but also embodied in visible and audible forms. Gaze provides direction to movement, vocalization and stomping provide pressure, and rhythm provides coherence. The idea is consistent with the theorizing in the sociology of emotion on emotion as an embodied process that is carried out through situational rules and social interaction (Hochschild, 1983) and with the perspective of embodied practice, where emotion is not only experienced within an individual, but also displayed and/or recognized through bodily actions (Grandey, 2000; Tamminen et al., 2024).

*The movement is done, but the rhythm is bad, and the point of force production cannot be found, so the force does not come through.*

*With something like Tai Chi, the movements may look in place, but the breathing rhythm and internal feeling are a bit disordered.*

These expressions suggest that the contextual aggressive state is not simply added to outward movement but is produced through the coordination of body, breathing, rhythm, and combat intention. Movements can be completed mechanically, but this state emerges only when these elements form a coherent whole.

### 4.3 Differences in the expression of contextual aggressive states across Wushu styles

The interview data also revealed that contextual aggressive states are not expressed in the same manner across different types of routines. The participants overall felt that Tai Chi focuses on intention, breathing and internal rhythm, while Changquan, Nanquan, animal-imitative forms and Xingyiquan focus on gaze, bearing, force production and combat meaning. One of the interviewees made the following remarks in a group interview:

*Tai Chi pays more attention to mind and intention, and its combat quality is not as strong. Nanquan and Changquan, by contrast, place more emphasis on bearing and gaze.*

Another participant, who had long practiced traditional routines, including animal-imitative forms, stated:

*I think that especially some traditional Wushu styles, such as Bajiquan and Xingyiquan, have a stronger aggressive state than standard Changquan, especially international Changquan and international Nanquan, because the standard routines have incorporated some gymnastics-like elements, which weaken this aggression.*

When discussing Heihuquan, he further emphasized:

*Heihuquan stresses bearing and gaze on the one hand, and on the other hand every movement in it has its own unique combat meaning. Gaze and the combat meaning of each movement together form this aggressive state.*

According to the interview data, the expression of contextual aggressive states in Wushu Taolu is shaped by style. In Changquan, there are more embodied expressions of these states through large, expansive movements and definite direction. In Nanquan, these states are more often expressed through vocalization, stomping, and a short, forceful sense of pressure. Traditional Wushu styles place greater emphasis on combat imagery, combat intention, and body method. Although Tai Chi does not use overt pressure, offensive and defensive meaning remains important, together with combat intention, breathing, and internal rhythm. That is, there is no fixed intensity profile for contextual aggressive states; rather, their expression depends on the requirements of a particular Wushu style.

These differences may be explained by the distinct training logics of different Wushu styles. In Taolu training, the contextual aggressive state is not a fixed personality trait but a stylized and context-dependent training state grounded in combat-oriented movement meaning. The findings show that this state is understood through its style-specific expression rather than through a uniform level of aggression.

## 5 From mental imagery to body-based cues: the active elicitation of contextual aggressive states and their instrumental significance

### 5.1 The active elicitation of contextual aggressive states through imagined opponents and combat intention

For some participants, entering a contextual aggressive state was not directly related to feeling angry; rather, this state had to be actively elicited during Wushu Taolu training. During the focus group discussions, participants were asked whether they still needed to elicit this state even when they were not actually angry; some gave a strong “Yes”:

*Yes. Many force-production methods in Xingyiquan require you to enter this kind of state.*

*The same is true of combat training and Nanquan. If you do not have this emotion, you will not have that sense of offensive intention.*

P2 also noted that he enters this state in training by “imagining an opponent”:

*P2: “I enter it by imagining an opponent and bringing in the situation. Usually, I first enter that kind of virtual offensive state internally, and then it is reflected outwardly to some extent.”*

P3 offered a more detailed explanation of this process:

*P3: “If I imagine having an opponent and train with intent to fight, as soon as I think of the situation, I'm focused and aggressive. If I practice a routine, I always ask myself, ‘What does this movement do? How is it used to attack?’ Only at this moment do I remember how the movement moves. Then I practice that same movement, I automatically imagine that situation.”*

These accounts do not suggest that the contextual aggressive state is directed toward actual conflict. Rather, imagining an “opponent” helps practitioners give direction, distance, and purpose to movement. Without an imagined target, movement may become mere posture; with such a target, it acquires combat meaning and combat intention.

This aligns with the instrumental emotion regulation perspective. Lane et al. (2011) suggested that, rather than allowing emotions to simply manifest beyond an athlete’s control, athletes might actively try to experience negative and/or high-arousal emotions because they believe these emotions aid performance. Similarly, both Jekauc et al. (2021) and Robazza et al. (2023) highlighted that emotions in sport cannot be categorized as positive or negative, but must be interpreted in the context of the task being performed. The contextual aggressive state described here may serve such an instrumental function in Wushu Taolu training. Although this state may not be appropriate in everyday interactions, during Taolu practice it can help students enter the combat-oriented logic of the movements.

### 5.2 Body-based cues: opening movements, muscle memory, and weapon sounds

Participants also noted that, in addition to mental imagery, bodily movements may help trigger contextual aggressive states. After extended training, some opening movements, shifts in gaze, weapon sounds, and modes of vocalization become “switches” for entering the state. P3 gave the following explanation:

*P3: “When you make the corresponding movements and expressions, once your eyes change and you perform the opening movement, muscle memory and the state formed through movement bring you into this state. The body unconsciously abstracts the memory of aggressive scenes into a movement symbol. When that movement symbol is performed, the body automatically recalls that aggressive state.”*

These accounts show that the elicitation of contextual aggressive states is not a one-way process from mind to body. It may begin with an imagined opponent and strengthened combat intention, but it may also begin with embodied cues such as gaze, opening movements, vocalization, and weapon use. One participant in the group interview described it in more detail:

*Sometimes the inner feeling comes first, and then the facial expression and posture follow. Then the facial expression and posture, in turn, strengthen that inner feeling. For example, just doing the opening movement can create that inner feeling, although it is not strongest at that point. As you continue the routine, the movements, facial expression, posture, and gaze then reinforce the inner feeling again, until it reaches its peak.*

This process involves mutual reinforcement between internal experience and embodied expression. The state is continually strengthened through bodily movement, mental imagery, muscle memory, and situational feedback. This phenomenon can be understood from the differences between surface acting and deep acting (Grandey, 2000), and Gross's (2015) understanding of emotion regulation processes.

Weapons and sound also function as triggers of this state. One participant in the group interviews said:

*If it is a weapon routine, shaking the weapon a little and hearing the sound can also raise your level of excitement.*

Another participant noted:

*It is a requirement in Nanquan to produce force and vocalize, and the angry sounds produce excitement naturally within Nanquan, which can bring you into the state.*

Thus, Taolu training is not an exclusively psychological process for eliciting contextual aggressive states. Weapon sounds, stomping, vocalization, and opening movements may function as embodied cues that shift students from an ordinary state into a performance-oriented contextual aggressive state. The results support the perspective of instrumental emotion regulation. Therefore, emotion regulation in Wushu Taolu operates in both directions: imagery influences embodied expression through a top-down process, while embodied expression, sound, and muscle memory influence imagery through a bottom-up process.

### 5.3 Performance enhancement and the risk of excessive performance-related arousal

Most participants perceived moderate contextual aggressive states as beneficial for Taolu training. They thought that these states could help focus attention, improve overall bearing, deepen their understanding of movements, and enhance stylistic presentation. One participant in the group interview said:

*It makes your attention more concentrated and helps you immerse yourself more deeply in the training state.*

P2 also noted:

*P2: “When you enter this state in training, the excitement generally makes you feel less tired. And with this state, you can also better understand the movement and know why that movement exists.”*

P1 mainly linked this effect to bearing and style:

*P1: “The most obvious effect is on the expression of bearing, and second is stylistic presentation. I do not think movement completion is very closely related to inner state, because movement completion requires long-term practice. But bearing and stylistic presentation are very clearly affected. When your state is there, when you can imagine and feel it, then you can present the corresponding bearing and style.”*

The instrumental value of contextual aggressive states is not directly related to the ability to complete a movement, but to whether the movement is performed with spirit, direction, and style. Long-term technical training is necessary for movement completion, whereas the contextual aggressive state gives the movement clearer combat intention and stylistic expression. This is consistent with the view that athletes consciously mobilize emotions to aid their performance (Beatty and Janelle, 2020; Jekauc et al., 2021; Robazza et al., 2023).

At the same time, the interview data show that a stronger state is not necessarily more beneficial. P3 recalled an experience during a physical performance examination:

*P3: “I became more aggressive than usual during the physical exam, got a bit too carried away—and, of course, sustained a sports injury. The movements were quicker than usual, the gaze more intense, and the rhythm faster than usual.”*

He further explained that excessive excitement can disrupt rhythm allocation:

*If you get overly excited, the early parts of the movements will be too fast initially, and later, you will get fatigued, so coaches often remind you to control the rhythm and not to become overexcited.*

These accounts show that the state has both functional benefits and potential risks. A moderate contextual aggressive state may support performance, whereas excessive performance-related arousal may disrupt movement control, accelerate rhythm, cause uneven energy distribution, and increase the risk of injury. Therefore, this state needs to be regulated within an appropriate range rather than intensified without limit.

## 6 From aggressive experience to aggressive desire: boundary regulation and the limited perceived transfer of contextual aggressive states

### 6.1 Distinguishing legitimate aggressive experience from excessive aggressive desire

The most theoretically significant distinction in the interviews was between what participants described as “aggressive experience” and “aggressive desire.” Private feelings, combat-related imagery, and performance states during training were regarded as legitimate aggressive experiences, whereas an actual desire to hit or harm another person was regarded as excessive aggressive desire.

*P1: “Aggression in martial arts is actually a kind of private aggression. Its degree lies in whether it is an aggressive feeling and aggressive imagination, or whether it turns into aggressive desire. When you practice martial arts, the movements are aggressive and have offensive intention, but you do not have the desire to attack. You are using your body to feel and recall that kind of attacking scene, rather than thinking, ‘I am practicing this routine because I want to hit someone.’”*

When asked what kind of expression should be considered excessive, P1 replied:

*P1: “Once there is aggressive desire, it becomes excessive.”*

Similar judgments also appeared in the group interviews:

*After practice, if you feel like you want to hit someone, then it has already crossed the line.*

*If it affects other people, then I think it has crossed the line.*

The participants did not deny the presence of contextual aggressive states in Taolu training. Rather, they explained that these states should remain at the level of emotion, combat-related imagery, and performance-related movement meaning. However, when imagined combat develops into an actual aggressive impulse directed toward others, it no longer constitutes a legitimate training state.

This finding helps to explain why previous research on martial arts training and aggression is mixed. Previous research tended to examine the changes in aggression caused by training, or whether it actually increased aggression levels. The present study, however, indicates that the question should not be whether aggression simply exists, but what form contextual aggressive states take among university students majoring in Wushu Taolu. More recent studies of emotional processes in sport have also indicated that aggression, anger, and excitement can form part of embodied arousal within specific rules and relationships, although they need to remain within appropriate limits (Tamminen et al., 2024; Kyed and Damsgaard Thomsen, 2025). In Wushu Taolu training, a contextual aggressive state may be perceived as functional, but it must remain bounded. This distinction moves the boundary question beyond the amount of aggression present.

### 6.2 The joint regulation of coaches, peers, and martial ethics

Participants attributed the perceived boundary between legitimate aggressive experience and actual aggressive behavior to multiple regulatory mechanisms within training. Their accounts indicated that coaches, peers, self-awareness, and martial ethics jointly regulated the boundary between contextual aggressive states and actual aggressive desire.

With regard to coaches, P2 noted that they both elicit the state and control excessive excitement:

*P2: “During typical training, people may be a little lazy and get tired; the coach may make use of this state to enhance the training condition. However, in competitions, because of the pressure already present, the coach may ask you to take deep breaths or speak to you, to decrease the pressure and reduce this state.”*

The group interviews also showed that if someone’s state became too strong, they would usually be reminded to stop:

*They can remind him to stop and rest for a while.*

*Very often, in the moment, there is this self-awareness: ‘I'm beginning to get over-excited about it,’ and that's when you start regulating yourself accordingly.*

These practices establish boundaries between an “insufficient state” and an “overly strong state.” When the state is weak, coaches elicit it through verbal cues, movement, and performance demands. When performance-related arousal becomes excessive, they use rhythm reminders, pauses, breathing techniques, and verbal guidance. Tamminen et al. (2024) maintained that emotion regulation in sport does not occur independently of coaches, peers, team norms, or relational contexts. The present data also highlight that contextual aggressive states in Taolu are continuously elicited, encouraged, and limited within the training community. A deeper layer of regulation comes from martial ethics. P3 stated:

*P3: “When you are learning martial arts, the first thing you should learn is martial ethics—it's the most important part of martial arts—and the fist-and-palm salute is also martial ethics. No matter how you phrase it, martial arts are always going to be accompanied by some level of aggression, and martial ethics should limit that aggression.”*

P1 recalled the recitation rituals in his martial arts school:

*P1: “In the past, before class, there used to be a discipline of martial ethics and rules for the training hall. They would also be posted before and after training. At the end of the day, after everyone was very tired, we would have to take a horse stance again and recite them loudly. This was very educational even though it seemed like formalism, because it would be repeated over and over again, and we would gradually take the words to heart; it was very instrumental in shaping emotion and temperament.”*

This endeavor demonstrates that martial ethics is not merely a slogan. A combination of ritualized training, repeated utterances and physical exercise makes these principles part of students’ behavior. The role of traditional martial arts in helping to lower anger levels and aggressive behaviors has also been explored in previous studies, suggesting that the etiquette, discipline and moral education provided during traditional martial arts instruction could be beneficial (Harwood et al., 2017; Lafuente et al., 2021). The present study also suggests that contextual aggressive states are embedded within martial ethics and structured training and are therefore bounded, restrained, and regulated.

### 6.3 From short-term residual arousal to long-term stylistic influence

The opening question, “Might it carry over?”—that is, “Does the contextual aggressive state developed in training extend into everyday life?”— initially elicited negative responses from several participants. When asked whether this state would carry over to dormitory life, school, interactions with friends, or online interactions, most participants in the group interviews shook their heads or responded, “Basically not.” One participant even said:

*The aggression in Wushu Taolu is mainly expressed in Wushu Taolu itself. It cannot appear in everyday life. If it did appear in everyday life, then martial arts would have been practiced for nothing.*

Direct behavioral transfer was not commonly reported in the interviews. Participants tended to distinguish training states from everyday-life states, while martial ethics also played an important role in this distinction.

However, more in-depth questioning revealed additional, less direct forms of transfer beyond the training context. The first was short-term residual arousal after training. One participant in the group interview said:

*After training, the body has not completely calmed down yet. You may feel emotionally elevated, but not very aggressive—just excited and rather aroused.*

Another participant added:

*Because you are in a highly aroused state, your reactions may also be quicker than when you are calm.*

P2 also noted:

*P2: “There may be a slight change in the emotional activation threshold. It may drop a little, making you more easily excited.”*

Participants described these effects as excitement, quicker reactions, and shifts in emotional activation thresholds. These accounts are consistent with residual arousal and possible cross-contextual influence. In line with Zillmann's (1971, 1983) excitation transfer theory, emotional arousal from one context can carry over to a subsequent context and influence later emotional responses. One participant in the group interview stated:

*After learning martial arts, we are naturally regarded as martial people, implying that our behavior in the classroom and with friends, as well as online, differs.*

Yet this long-term influence does not necessarily mean becoming more aggressive:

*People who practice martial arts may actually become less aggressive. Coaches always say that martial arts begin with moral cultivation. When we encounter a situation of real anger, we may be more aware of the consequences of using violence, and our aggression may actually be lower.*

This suggests that the perceived effects of training states in everyday life may operate in two directions. On the one hand, martial arts experience may make a person appear more forceful, more direct, or more “martial” in the eyes of others. On the other hand, long-term training and martial ethics education may strengthen self-control, making students more aware of the consequences of violent behavior and thus less likely to engage in real aggression. This finding is broadly consistent with Huang et al. (2026), who reported lower off-field aggression among competitive athletes than non-athletes. The present study may complement this evidence by suggesting that coach regulation, peer norms, martial ethics, and boundary awareness may help prevent contextual aggressive states from developing into actual aggressive desire.

Kendellen and Camiré (2019) argued that sport-to-life transfer is not an automatic carryover of sport experience into life, but a process in which abilities, attitudes, and behavior patterns developed in sport are transformed and applied outside sport under certain conditions. Tanious et al. (2023) likewise showed that sport experience may enter learning, interpersonal relationships, and social life, but such transfer is shaped by individual differences and contextual conditions. Participants’ accounts did not indicate direct behavioral spillover of contextual aggressive states, but suggested that some associated effects might extend beyond training. Participants described short-term residual arousal, longer-term identity-related influence, strengthened self-control, and, in some cases, the continuation of confrontational emotions. Their accounts therefore indicate that direct behavioral transfer was not commonly perceived, while some participants reported possible indirect effects.

### 6.4 Individual differences in perceived transfer: personality, martial ethics education, coaching style, and Wushu style experience

Another important finding concerned whether and how contextual aggressive states or their associated effects were perceived to transfer, and these patterns varied considerably across individuals. The participants mostly thought that personality, family education, developmental environment, martial ethics education, coaching style, competition experience and Wushu style would influence this process.

P1 claimed that, since the training environment was kept the same, the significant differences arose from personal backgrounds:

*P1: “If you control the variables—same age, same training hall, same teacher, same teaching method—some people are more likely to bring the training state into life, while others are not. The three factors are family teaching, personality and the overall development environment. The more rational a person is, the less likely they are to bring their psychological state into everyday life.”*

P1 further noted:

*P1: “Assuming the rationality is similar, whether they bring training state into life depends on martial ethics education and training hall education.”*

P2 also believed that transfer is related to personality, martial ethics education, and coaching:

*P2: “A person’s personality, martial ethics education, self-control, and the coach’s instruction are all important.”*

In addition, participants suggested that greater competition experience might be associated with lower aggression. P2 stated:

*P2: “Competition experience matters too. The more competitions you see and the more experience you gain, the more you reflect on yourself and realize you are not that great, and then aggression may actually become weaker.”*

The type of Wushu discipline and training also makes a difference. In the group interviews, one participant pointed out that confrontational training differs from Taolu training:

*Those who practice Sanda are usually more aggressive, because real confrontation naturally produces a kind of fierceness. If they cannot control it well, their aggression may become stronger. For those of us who practice traditional Wushu Taolu, we may appear more aggressive or more withdrawn in the eyes of others, but that does not necessarily mean we are truly more aggressive.*

These accounts suggest that there may be more than one mechanism underlying the perceived transfer of contextual aggressive states and their associated effects. Such transfer may be shaped by the interaction of training content, personality, coach regulation, and martial ethics education. Some Wushu styles or forms of training may strengthen overall bearing and increase performance-related arousal, but whether these effects extend into actual conflict may depend on individuals’ boundary awareness and self-control.

Based on these findings, it may be reasonably inferred that responses to situational triggers are shaped by both residual arousal and regulatory mechanisms. After intensive training, quicker reactions and changes in emotional activation thresholds may become more noticeable and may influence responses to interpersonal conflict in classrooms, dormitories, or public settings. However, the present self-report data cannot determine whether these effects result in actual aggressive behavior.

From an educational perspective, Wushu Taolu training should not simply seek to suppress contextual aggressive states, but should guide students to understand their contextual boundaries. These states are related to combat meaning and stylistic expression and cannot be entirely removed from training. However, excessive performance-related arousal and aggressive desire should be regulated through coach guidance, training discipline, peer norms, and martial ethics education. Kyed and Damsgaard Thomsen (2025) similarly noted that sport may involve excitement, aggression, and anger, but these emotions must be regulated to prevent interpersonal problems. Accordingly, contextual aggressive states may form part of Wushu Taolu training, but they should remain within combat-related imagery and stylistic expression rather than develop into inappropriate behavior toward others.

## 7 Conclusion

The objective of this study is not merely to determine whether Wushu Taolu training increases or decreases aggression. Rather, it reveals the specific forms and meanings of contextual aggressive states that appear in Taolu training. The findings indicate that the phenomenon identified by participants should be interpreted as a contextual training state grounded in the offensive and defensive meanings of movement, rather than as hostility or violent impulses associated with actual physical aggression. Participants recognized this state when a movement acquired a clear target, direction, combat intention, and stylistic expression. Thus, the contextual aggressive state identified in this study is embedded in movement, combat-related imagery, and Taolu performance.

This study also revealed two pathways for eliciting contextual aggressive states: mental imagery and body-based cues. Students can enter this state by imagining an opponent and strengthening combat intention, and may also do so through opening movements, shifts in gaze, weapon sounds, vocalization, and muscle memory. Thus, Taolu training is not merely a process of mentally representing combat intention or producing embodied expression; it is also a process through which mental imagery and bodily action jointly construct a contextual aggressive state.

The study also indicated that the crucial issue concerning contextual aggressive states is not simply their intensity, but whether participants experienced them as an “aggressive experience” or an “aggressive desire.” The former remains within the realm of combat-related imagery, performance states, and movement meaning and is therefore regarded as a legitimate training state. The latter involves an actual aggressive impulse or an intention to attack or harm others and therefore constitutes an excessive state. The boundary between these states is regulated through coaches, peers, self-awareness, and martial ethics.

Participants generally did not perceive direct behavioral transfer of contextual aggressive states from training into everyday life. However, they described more limited associated effects, including short-term residual arousal, quicker reactions, altered emotional activation thresholds, and identity-related or stylistic influences following training. Because these findings were based on participants’ self-reports, the study cannot determine whether behavioral transfer objectively occurred. It can only indicate that direct behavioral transfer was not commonly perceived, while some participants reported possible indirect effects.

## 8 Limitations and future directions

This study has several limitations. First, the findings were based on retrospective self-reports and may have been influenced by social desirability. Although the moderator encouraged independent and divergent responses, conformity and mutual influence could not be completely excluded. Therefore, the study cannot determine whether behavioral transfer objectively occurred. Second, because Wushu Taolu is a prearranged and non-contact practice and no physiological or behavioral measures were collected, the findings cannot be generalized to actual physical confrontation. The single-university sample also limits the transferability of the findings.

Future studies should compare participants from different institutions and training backgrounds and combine interviews with longitudinal, behavioral, and physiological evidence. Research should prioritize specific aggression-related dimensions, including anger, hostility, violent impulse, actual aggressive behavior, and emotional activation thresholds. It should also identify the situational conditions most consistently associated with residual arousal or confrontational expression, examine how Wushu Taolu training influences responses to these triggers, and evaluate whether coach guidance, martial ethics education, post-training recovery, and non-violent conflict-management training can help reduce or manage aggression-related triggers in university and public settings.

## Data Availability

The original contributions presented in the study are included in the article/supplementary material, further inquiries can be directed to the corresponding author.
